# Determinants of male involvement in maternal and child health services in sub-Saharan Africa: a review

**DOI:** 10.1186/1742-4755-9-32

**Published:** 2012-11-21

**Authors:** John Ditekemena, Olivier Koole, Cyril Engmann, Richard Matendo, Antoinette Tshefu, Robert Ryder, Robert Colebunders

**Affiliations:** 1 Elizabeth Glaser Pediatric AIDS Foundation, Kinshasa, Democratic Republic of Congo; 2 Institute of Tropical Medicine, Antwerp, Belgium; 3 University of North Carolina at Chapel Hill, North Carolina, USA; 4 Kinshasa School of Public Health, Kinshasa, Democratic Republic of Congo; 5 Division of Hospital Medicine, University of California, 200 West Arbor Drive #8485, San Diego, USA; 6 University of Antwerp, Antwerp, Belgium

**Keywords:** Male involvement, HIV/AIDS, MCH services

## Abstract

**Introduction:**

Male participation is a crucial component in the optimization of Maternal and Child Health (MCH) services. This is especially so where prevention strategies to decrease Mother-to-Child Transmission (MTCT) of Human Immunodeficiency Virus (HIV) are sought. This study aims to identify determinants of male partners’ involvement in MCH activities, focusing specifically on HIV prevention of maternal to child transmission (PMTCT) in sub-Saharan Africa.

**Methods:**

Literature review was conducted using the following data bases: Pubmed/MEDLINE; CINAHL; EMBASE; COCHRANE; Psych INFORMATION and the websites of the International AIDS Society (IAS), the International AIDS Conference and the International Conference on AIDS in Africa (ICASA) 2011.

**Results:**

We included 34 studies in this review, which reported on male participation in MCH and PMTCT services. The majority of studies defined male participation as male involvement solely during antenatal HIV testing. Other studies defined male involvement as any male participation in HIV couple counseling. We identified three main determinants for male participation in PMTCT services: 1) Socio-demographic factors such as level of education, income status; 2) health services related factors such as opening hours of services, behavior of health providers and the lack of space to accommodate male partners; and 3) Sociologic factors such as beliefs, attitudes and communication between men and women.

**Conclusion:**

There are many challenges to increase male involvement/participation in PMTCT services. So far, few interventions addressing these challenges have been evaluated and reported. It is clear however that improvement of antenatal care services by making them more male friendly, and health education campaigns to change beliefs and attitudes of men are absolutely needed.

## Introduction

Prevention of mother to child transmission (PMTCT) of Human Immunodeficiency virus (HIV) infection should be prioritized in sub-Saharan Africa [1]. Barriers hindering uniform implementation of this highly successful prevention strategy need to be identified and addressed. According to the World Health Organization (WHO), the Joint United Nations Programme on HIV/AIDS (UNAIDS) and the United Nations Children’s Fund (UNICEF) an estimated 390,000 infants contracted HIV during the perinatal and breastfeeding period in 2010 [1]. Nearly all these infections, in principle, should have been prevented. The most common route of transmission for these infants is transmission from mother to child (MTCT) which occurs in up to 90% of cases [1]. In sub-Saharan Africa, women comprise more than half the number of people living with HIV and the majority of these HIV positive women were infected by their stable partners [2-8]. Since husbands play a pivotal role in decision-making within the home, and are often the main bread winners, establishing their buy-in and support for PMTCT activities and interventions is critical [9-14]. A husband’s role is a likely determinant for the successful implementation of PMTCT guidelines/standards in Sub-Saharan Africa [15,16].

Male participation in child-bearing decisions is crucial and also has a positive impact on the acceptability of PMTCT interventions [17-24]. Providing suitable medical information to men has several important consequences related to PMTCT interventions [18]. First, well-informed men will be more likely to participate positively in the decision making for the well-being of the couple [25,26]. Second, women with supportive partners will be more motivated to undergo HIV testing, to return for the HIV test result and to disclose the HIV result to their partner [19,23]. Third, well-informed couples may be more likely to adopt a low risk behavior and increase mutual support, regardless of the test result [27-29]. Studies have shown that in countries with high HIV prevalence there is also a high incidence of HIV infection in women during pregnancy or in the post-partum period. Indeed in this period women are particularly vulnerable to become HIV infected [30-32]. Therefore it is very important that partners of pregnant women are also tested for HIV and that antiretroviral treatment is considered if they are found to be HIV infected [30-41]. Fourth, decisions regarding the choice of a family planning method as well as the newborn feeding method can be made together [14]. Finally, if an HIV positive mother is pregnant and eligible for Antiretroviral Treatment (ART), she should start treatment as soon as possible. If she is not eligible for ART, antiretroviral (ARV) prophylaxis needs to be initiated as early as 14 weeks of gestational age [15,16]. Thus male involvement is very likely to lead to better adoption of HIV prevention practices by a well-informed couple [30-41].

There is also a strong inverse relationship between low male participation in PMTCT services and high MTCT risk in exposed infants. A study conducted in Nairobi/Kenya between 1999 and 2005 found that MTCT risk in exposed children was significantly associated with low male participation in Maternal and Child Health (MCH) services. In women whose male partners had come to the antenatal care (ANC) clinic, there was less MTCT compared with women whose partners did not take part in the PMTCT interventions (aHR =0.52; 95% CI: 0.32 - 0.84; p=0.008) [17]. Male involvement in PMTCT improves ARV prophylaxis uptake, adherence and promotes compliance for family planning, and optimal infant nutrition [5,21,22].

The objective of this paper is to review the literature about determinants of male partners’ involvement in MCH activities, with a focus on PMTCT services in low-income countries, specifically sub-Saharan Africa.

## Methods

### Participants, interventions and outcome

Participants in this review were male partners of pregnant women attending antenatal and under five clinics. The male partner may be the baby’s father or not. Our research focused on interventions tailored to have an impact on PMTCT, HIV counseling, couple counseling, reproductive health education, family planning and safe delivery. The outcome of this review was male involvement in these interventions.

### Search strategy

The following electronic data bases were used to identify the articles: Pub med/MEDLINE; CINAHL; EMBASE; Cochrane Library and Psych INFO. We limited our search strategy to articles published between January 1990 and October 2011. The websites of the International AIDS Society (IAS), the International AIDS Conference and the International Conference on AIDS in Africa (ICASA) 2011, WHO, UNICEF and UNFPA were used to find relevant abstracts and documents.

Search terms consisted of the following key words: “HIV testing”; “prevention”; “mother”; “child”; “male partner *”; “counseling”; “involvement”; “participation”, sub-Saharan Africa”. And the grouped terms“ PMTCT and partners”; “VCT and acceptability in PMTCT”; “barriers and/or factors”;“ Male involvement in PMTCT”; “Male involvement in reproductive health”.

### Screening and papers selection criteria

The first screening round of publications was carried out based on the titles. The second screening round of the remaining papers was conducted using the abstracts. In the final round, the remaining publications were assessed using the full texts.

The following criteria were used to exclude ineligible papers:

– studies not addressing the issue of determinants of male involvement in PMTCT;

– studies not conducted in sub- Saharan Africa;

– published in languages other than English;

– comments, debates, reviews, personnel opinions;

– theses and dissertations;

– reports of activity implementations;

– studies published before 1990;

– papers related to the tools/instrument developments;

### Data extraction

Data was extracted from the full texts and abstracts. The extracted information consisted of: authors, year of publication, research question, study settings, purpose and study objectives; study design, study population, participants number, participants type, interventions type, study outcomes, study results, male participation barriers, male participation factors, male participation definitions, study timeline and study limitations.

## Results

### Search flow

**Figure 1:**
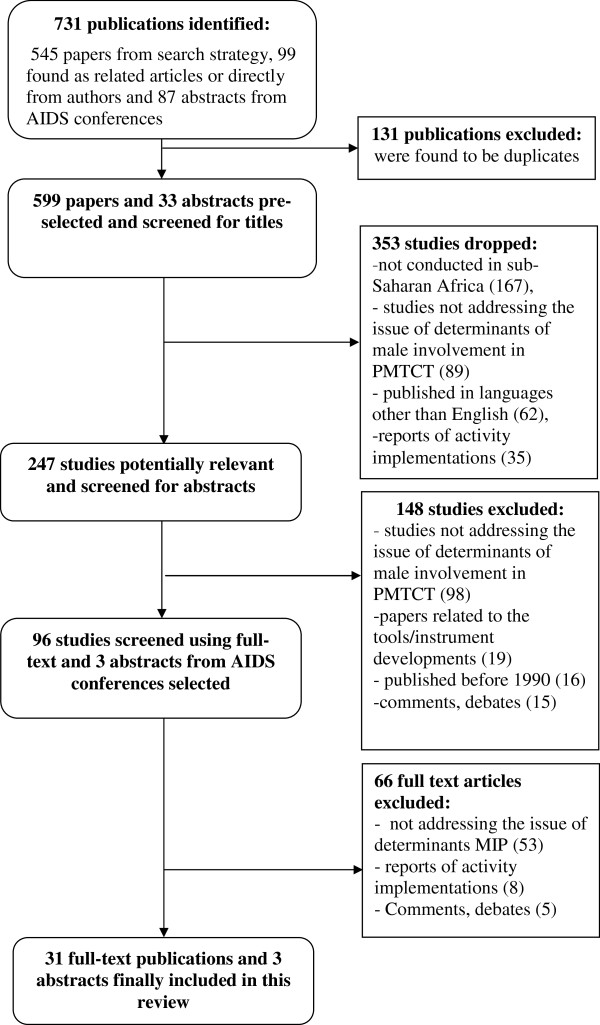
Search flow.

The reviewers identified 731 publications, 132 of them were duplicates. After first and second rounds of screening of 599 remaining publications based on the titles and abstracts, 99 studies were pre-selected for the final screening using the full-text. At the end of final review and assessment, 34 eligible studies were included in this review. Details related to the search flow are included in the Figure 1.

### Concept and definitions: male involvement and male participation

A precise and universally accepted definition of male involvement in PMTCT is lacking. The definition of the term “male involvement” varies according to authors. Some authors define male involvement as male partners’ participation in HIV testing solely during ANC [9,10,14]. Others consider participating in couple counseling as male involvement [9,11-13]. In this paper we will use the composite term “Male Involvement and Participation” (MIP).

### Male involvement/participation factors

In our review we identified three categories of factors associated with male involvement/participation (MIP):

1) Socio-demographic factors

A. *Age and marital status*: Most studies reported that older age and cohabiting were associated with male involvement [8,10-13,42-44]. Our group conducted a study in Kinshasa and found male involvement was 1.2 times higher among men whose female partners were 25 years or older. Monogamous partners and co-habiting men were twice and 1.6 times respectively more likely to be involved [10]. In contrast, Nkuoh et al. reported that Cameroonian men in polygamous relationships showed higher involvement [13].

B. *Education*: A study in Uganda found that men who had completed 8 or more years of education were twice more often involved compared with those with less than 8 years of education (OR =1.9; 95% CI: 1.1-3.3; p≤ 0.05) [11]. This was not confirmed in our study in Kinshasa where the level of education of pregnant women or their male partner did not influence male participation [10].

C. *Profession*: In Uganda, taxi drivers and “Bodaboda” riders (motorbike taxi riders) were less likely to participate than men with other professions such as farmers or construction workers (OR =0.3; 95% CI: 0.1-0.9; p≤ 0.05) [11]. Other authors have corroborated these findings. Reece et al. reported that Kenyan men having only an occasionally job were less likely to participate in MCH services [12]. Another study from Rwanda reported that men with a well-paid job were more likely to participate in PMTCT interventions compared to those not well paid [45].

2) Health service- related factors

A. * Harsh, critical behavior and language use*: Byamugisha et al. reported that harsh, critical language directed at Ugandan women from skilled health professionals was a barrier to male participation [11]. Harsh treatment of men by health providers discouraged them from returning or participating in PMTCT activities [11]. Furthermore, some providers did not allow men access to ANC settings [11].

B. *Financial constraints*: Financial constraints of clients and health facilities have been identified as impacting health services uptake and male participation [3,12-14,46,47]. A Ugandan study reported that some health providers charged extra beyond the official ANC fees to bridge their own financial gaps [11] while other authors have identified low health providers’ salaries as limiting factors for male involvement [47,48].

C. *Venue and space constraints*: In our study in the DRC, men were invited for voluntary counseling and testing (VCT) in three venues: a bar, a health center or a church. Male involvement in VCT was higher in the bar (26, 4%, p < 0,001) and church (20,8%, p = 0,163) compared with the health center (18,2%) [10]. These results suggest that more friendly and convenient venues for men are needed [19]. The lack of space to accommodate male partners in ANC clinics was also reported to adversely impact male involvement [11]. Clinics are often unable to concurrently accommodate pregnant women and their partners because of a lack of space. Gender specific services to address uniquely male issues do not exist. Targeted interventions for men, such as tailored messages, specific health education sessions, and innovative strategies to identify male friendly venues would be valuable for increasing male involvement [10].

D. *Waiting time*: Frequently women have to wait for a long time before receiving ANC services because of burdensome administrative procedures which result in poor patient/client through-put in health facilities. Men, who frequently are in the paid workforce, are often not in a position to spend virtually the entire day participating in ANC services [11].

E. *Quality of care*: In a study in Rwanda it was shown that essential PMTCT services were often not proposed by health providers thus contributing to the weak PMTCT ARV prophylaxis uptake among clients [21]. Health services providers are often overworked, stressed, and have to work in an infrastructure with severely limited resources. In such context, the quality of services is compromised and taking care of participating male partners is considered an additional burden [47,48].

F. *Time of day for providing PMTCT services*: Increased male participation in VCT and couple testing occurred in Kinshasa when the MCH services are open in the evenings between 5:00 – 8:00 pm and at weekends [10]. Most health facilities offer these services only on weekday mornings, when the majority of men are at work. Yet several studies have identified ANC opening hours as a limiting factor for male involvement [13,14,48]. Permanent PMTCT services would facilitate the services’ uptake even for men with difficult work schedules [10,12]. Geographical constraints impact health services uptake and male participation [3;9;12–14;47]. Lack of decentralized services is a reason for low health services uptake and limited male involvement [48]. A qualitative study conducted in western Kenya by Reece et al. found that the distance that the male partners have to travel to the clinics for participating in the education, HIV tests and counseling, the costs of the transport to the clinics and the amount of time per appointment at the clinic were identified as barriers to male involvement [12]. Data from another study from Uganda showed that majority of participants said that the health facilities were few and located far from the people, making the health services such as HIV testing and counseling inaccessible [48]. Most of the male partners and men in general wanted the health services to be implemented and extended to their villages or close to their homes in order to save them the costs of time and travel fee [48].

3) Sociologic factors

A. *Cultural*: In several studies cultural standards were identified as barriers for male involvement [11-14]. Several studies have reported negative perceptions towards men attending ANC services. In one report, men who accompanied their wives to ANC services were perceived as being dominated by their wives. Frequently men perceive that ANCs services are designed and reserved for women, thus are embarrassed to find themselves in such “female” places [11,26,27]. Certain women too, do not like to be seen with their male partner attending the ANC service [12,26]. A study conducted in Kenya showed that certain male clients trust traditional healers but not hospitals and therefore do not attend ANC clinics [12].

B. *Male attitudes and beliefs*: Fear of receiving an HIV positive result and confidentiality concerns prevent some men from coming for VCT. In many studies men were mentioned being concerned about HIV-associated stigma and disclosure [12,49,50]. Men may be afraid of HIV status disclosure in a health system facility, in the context of weak health system [51].

C. *Female attitudes and considerations*: Several studies showed that women at ANC clinics fear violence from their partners who attend ANC clinics with them. These women fear that discovery of a positive HIV test result may lead to abandonment, rejection or being perceived by their husband as being responsible for bringing HIV into the couples’ relationship [18,39-41,44,52]. Gender-based violence is another cause of low male involvement [18,42,49,53,54]. Victims of gender-based violence may be afraid to ask their partner to be tested for HIV. Reinforcement of women’s’ power for negotiation would be a major asset [14,55]. Msuya reported from the study conducted in Tanzania that male partners of women with higher income were more likely to participate in HIV testing and counseling. Also, women with higher education were more likely to have discussed HIV and reproductive health issues with their male partners (94.3% versus 88.3%; p<0.001) [14]. Alcohol use was identified as another factor for non-participation of men [27,44-54]. Daily overconsumption of alcohol by male partners maybe particularly implicated as a catalytic event for physical violence towards women. In similar regard, Karamagi reported alcohol as one of reasons for 54% of lifetime partner’s violence and 14% of physical violence in Uganda [52]. Ntanganira found the 35.1% of intimate violence in the last year; physical violence was twice likely to occur if a woman was HIV positive than negative [44].

D. *Communication*: Poor communication between men and their female partners was associated with poor male involvement. On the other hand, good couple communication was associated with high HIV status disclosure and support between husband and wife [12].

## Discussion

This review showed that different definitions of male involvement in PMTCT are used in different studies resulting in difficulties when comparing data between these studies. Determining a consensus definition of male involvement may be a necessary first step to measure efficacy and enhance comparability across programs [16]. In most of the studies we reviewed male involvement was considered as male participation in HIV testing during ANC. Other studies considered male involvement as male participation in HIV couple counseling.

Some authors classify MIP in two categories: “positive MIP” and “negative MIP” [19,39-42]. “Positive MIP” increases the engagement of women in PMTCT activities [19,36-40]. Positive MIP includes discussing HIV testing with the partner, being supportive regardless of the HIV result, participation in couple counseling and willingness to accompany the pregnant women to the ANC [18,19,30,31]. “Negative MIP” includes violence towards the partner, not discussing HIV testing with the partner and even prohibiting the partner to be HIV tested [19,39-42].

Byamugisha et al. scored male involvement using 6 variables: the male partner accompanying his wife during ANC services; knowing the ANC schedule; discussing the ANC interventions with the female partner; supporting the ANC fees; knowing what happens at the ANC; and using a condom with the female partner during the current pregnancy. Scores between 0–3 were considered weak male involvement and scores of 4 and above were considered as high male involvement. While this scoring system is a useful first step, it remains to be validated [11].

We speculate that adoption of a uniform definition of MIP and further studies specifically focused on metrics assessing male involvement in PMTCT services will be useful tools for monitoring and evaluation of HIV and MCH-related programs and research.

Most studies reported that older age, cohabiting and monogamy were associated with male involvement [8,10-13,42-44]. An explanation for this could be that older men may have a higher risk perception and that cohabiting men and women may have more time to harmonize their time schedules and to communicate. It is unclear why polygamous men in Cameroon were more likely to be involved in MCH services [13]. A possible explanation is that such men by virtue of having more than one partner are invited more frequently to the health facility. An alternative explanation could be that they are more financially secure, and thus more able and willing to pay for and wait with their partners to receive MCH services.

Many explanations for provider harshness and lack of respectful care to patients have been suggested. These include provider low salaries, lack of a functioning health infrastructure and a critical shortage of health care providers [11]. While these are certainly realities working in sub-Saharan Africa, it is clear that further training in nursing, midwifery and medical schools on the principles of family-centered care, combined with improved customer care communications are urgently needed.

When there is limited physical space to accommodate male partners, providers will have difficulties incorporating male partners [11]. This situation is worsened when health care workers are understaffed, underpaid and overworked.

Given that the staffing and financial situations in many health care systems in sub-Saharan Africa are unlikely to improve overnight, alternative models of care, targeted at men, are imperative if men are to participate in MCH activities. These may include the following: implementation of systems improvement strategies to improve patient attendance and flow through the health system; use of an appointment system and/or letter of invitation by the health provider; broadening services to the evenings and weekends; and consideration of multiple venues not traditionally associated with health care provision such as bars, bus stops and churches [10,47,48]. Access to health services for male partners should be prioritized [56]. In addition, in order to maximize the PMTCT uptake, a family centered approach is important since others members of mother‘s family such as mother’s father, brother, brothers and others male friends also may have an impact on the PMTCT uptake. Actions should be taken as well to involve those peoples [57-61].

### Limitations of this review

Many of the studies were conducted in countries with a different cultural context and used different study designs. We speculate that a harmonized international study regarding the MIP would be more comprehensive and generalizable across countries.

## Conclusion

There are many challenges to increase male involvement/participation in MCH and PMTCT services. So far very few interventions addressing these challenges have been evaluated scientifically. Capacity reinforcement of health providers through training and adequate salary support is needed. Improving accessibility, affordability, availability, accommodation and acceptability (5 A’s) of ANC service venues will make them more attractive for male partners. Additionally, health education campaigns to improve beliefs and attitudes of men are absolutely needed.

## Competing interests

All the authors declare that they have no competing interests.

## Authors’ contributions

JD, OK, CE, RM, AT, RR and RC had significant intellectual contribution and input in the conception and design of this review, draft writing, and final approval of the manuscript. All authors read and approved the final manuscript.
